# Correlates of Aggression in Men With Methamphetamine Use Disorder: Childhood Trauma and Methamphetamine-Use Characteristics

**DOI:** 10.3389/fpsyt.2022.888055

**Published:** 2022-05-20

**Authors:** Mengqi Liu, Liujin Pu, Tieqiao Liu, Xiaojie Zhang, Hongxian Shen, Qijian Deng, Yingying Wang, Wen Li, Xiaoya Fu, Cheng Yang, Ting Fang, Marc N. Potenza, Wei Hao

**Affiliations:** ^1^Department of Psychiatry and National Clinical Research Center for Mental Disorders, The Second Xiangya Hospital of Central South University, Changsha, China; ^2^Department of Kidney Transplantation, The Second Xiangya Hospital of Central South University, Changsha, China; ^3^School of Physical Education and Health, Hunan University of Technology and Business, Changsha, China; ^4^Key Laboratory of Forensic Science and Ministry of Justice, China Are Affiliated to Academy of Forensic Science, Shanghai, China; ^5^State Key Laboratory of Toxicology and Medical Countermeasures, Beijing Key Laboratory of Neuropsychopharmacology, Beijing Institute of Pharmacology and Toxicology, Beijing, China; ^6^Department of Psychiatry, Yale University School of Medicine, New Haven, CT, United States; ^7^Child Study Center, Yale University School of Medicine, New Haven, CT, United States; ^8^Connecticut Council on Problem Gambling, Wethersfield, CT, United States; ^9^Connecticut Mental Health Center, New Haven, CT, United States; ^10^Department of Neuroscience, Yale School of Medicine, New Haven, CT, United States

**Keywords:** substance-related disorders, addictive behaviors, aggression, methamphetamine use disorder, childhood trauma, men, China

## Abstract

**Background:**

Aggression is common among individuals with methamphetamine use disorder (MAUD) and constitutes a serious public health issue. The current study aimed to examine associations of methamphetamine-use characteristics and childhood trauma with aggression in men with MAUD.

**Methods:**

This cross-sectional study was conducted from December 2017 to August 2018. MAUD patients recruited from a compulsory drug rehabilitation center (n = 360) and healthy comparison subjects (n = 604) completed a survey that measured aggression and childhood trauma, using the Chinese version of Buss-Perry Aggressive Questionnaire (AQ-CV), and the short form of Childhood Trauma Questionnaire (CTQ-SF), respectively. MAUD patients also provided information on methamphetamine-use characteristics such as the age of MAUD onset, MAUD severity, and co-occurring alcohol use disorder (AUD) and tobacco use disorder (TUD) using standard or self-designed questionnaires. Chi-square tests and *t*-tests were used to compare childhood trauma and aggression between the MAUD and comparison groups. Multiple linear regressions were conducted to determine correlates of overall aggression and its five sub-scales among the MAUD group.

**Results:**

The MAUD group had higher childhood trauma and aggression scores than the comparison group. Within the MAUD group, age of MAUD onset, having severe MAUD, co-occurring AUD, co-occurring TUD, and childhood trauma were associated with overall aggression, with slightly different correlates found for its five sub-scales.

**Conclusions:**

Our study shows a high level of childhood trauma and aggression in the MAUD group. Both methamphetamine-use characteristics (age of MAUD onset, severe MAUD, co-occurring AUD/TUD) and childhood trauma were associated with aggression in MAUD. Our findings provide useful information on potential risk factors for aggression and inform future longitudinal research to establish causal relationships between these factors and aggression to guide further prevention and treatment programs.

## Introduction

Methamphetamine use disorder (MAUD) has become a serious public health problem globally. According to the 2021 World Drug Report, roughly 27 million people (0.5% of the global adult population aged 15–64 years) were estimated to have used amphetamines in 2019, with methamphetamine (MA) being the most used amphetamine-type stimulant (ATS) in East and South-East Asia (1). The China Drug Trend Report 2019 shows that 2.148 million people have illicit substance use disorders (SUDs) and 55.2% (1.186 million) of them have MAUD, indicating that MA is the most abused illicit substance in China (2). According to the China Drug Trend Report 2020, although the COVID-19 pandemic may have created conditions for or prevented drug abuse, MA remains the main abused illicit substance in China (3).

Aggression has been well documented as a common phenomenon among people with SUDs, especially MAUD (4, 5). Aggression is defined as behavior that is elicited by provocation, driven by anger, and intended to harm another person (6). Aggression represents a state phenomenon or a disposition to behave aggressively across various situations and over repeated occasions (7). Aggression may take many forms, ranging from relatively minor acts (e.g., pushing) to severe acts (e.g., stabbing or killing). Aggressive cognitions (e.g., hostile attitudes, beliefs, or thoughts) and emotions (e.g., feelings of anger) often serve as important precursors to aggressive behaviors. The relationship between MAUD and aggression is complex and likely explained by a wide range of psycho-socio-biological factors, among which MA-use characteristics and childhood trauma have been studied and reported (8–10).

Several MA-use characteristics have been identified as affecting aggression among patients with MAUD, including age at first use of MA, duration and frequency of MA use, route of MA administration, psychotic symptoms, and combined use of other substances such as alcohol. Early age at first use of MA increased the likelihood of engaging in MA-related violence after adjusting for other factors and socio-demographics (11, 12). In addition, chronic MA use (13), more frequent MA use (14), and MA injection (15) were all linked to difficulties controlling anger and the exhibition of more aggressive behaviors. Furthermore, MA use may increase positive psychotic symptoms such as hallucinations and delusions, which may create a sense of danger or threat and thus lead to defensive or pre-emptive aggression (14, 16, 17). Therefore, positive psychotic symptoms may further strengthen the risk of aggression in people with MAUD (5). Finally, the combined use of other substances, particularly alcohol, is common in people with MAUD, especially in nightlife environments where aggressive behaviors often occur and have thus been associated with aggression (18, 19). In summary, MA-use characteristics, including early age at first MA use, chronic and frequent MA use, MA injection, positive psychotic symptoms, and combined use of alcohol, all may contribute to aggression among people with MAUD.

Compared to MA-use characteristics, childhood trauma has been less studied as a potential risk factor for aggression among patients with MAUD, although data indicate positive associations between childhood trauma and aggression in samples from prisons (20), colleges (21), and the general population (22). Childhood trauma involves any adverse experiences that may harm or threaten the health and welfare of a child under the age of 16 years and may include physical, mental, or sexual abuse, or neglect (23). Experiencing childhood trauma has been identified as a risk factor for both aggressive behaviors and substance use problems such as MAUD (24, 25). A recent review (26) demonstrated that experiences of childhood trauma were associated with SUDs (including MAUD), which in turn increased the likelihood of aggression and violence. These findings provide further evidence regarding the links between and mechanisms underlying childhood trauma and aggression among individuals with MAUD.

Despite some evidence documenting associations of MA-use characteristics and childhood trauma with aggression among individuals with MAUD, there have been several methodological concerns that may limit a comprehensive understanding of such associations. First, most studies focused only on individuals with MAUD and did not compare childhood trauma and aggression with a control comparison group. Second, most studies tended to treat aggression as a unidimensional construct, rather than as a spectrum of behaviors that range from verbal to physical and psychological aggression (27). Since most studies focused on only one aspect of aggression, it remains unclear whether certain dimensions may differ from others with respect to patterns of associations. Third, while the association between MA-use characteristics and aggression among patients with MAUD has been studied, relatively less is known about associations between childhood trauma and aggression among patients with MAUD. To our knowledge, no study has ever included both MA-use characteristics and childhood trauma as potential risk factors to examine their effects comprehensively and simultaneously on aggression among patients with MAUD. Fourth, most previous studies on aggression among patients with MAUD were conducted in Western countries, and less is known about situations in Asian countries like China, where MAUD has been a major issue with increasing prevalence.

In light of the research gaps identified above, we conducted the current study to compare childhood trauma and aggression in MAUD patients and healthy control subjects, to examine associations of MA-use characteristics and childhood trauma with aggression in MAUD patients. This study addresses the above-mentioned limitations of previous studies by adding a healthy control group, including a broader measurement of aggression to assess its various dimensions, examining both childhood trauma and a range of MA-use characteristics, and involving a sample of male patients with MAUD recruited from a compulsory drug rehabilitation center in China.

## Method

### Participants and Procedures

Participants in the MAUD group were conveniently sampled from a compulsory drug rehabilitation center in Changsha, China, between December 2017 and August 2018. The inclusion criteria for the MAUD group were: 1) male (since aggression is much more common among male MAUD patients, and all MAUD patients in the study site were male), 2) age ranging from 18 to 60 years, 3) at least 14 days of detoxification at the time of study participation to avoid the negative effects of acute MA withdrawal symptoms (usually peaks within 24 hours after MA abstinence and resolves over the next two weeks) on data collection quality, 4) the core illicit substance abused was MA, and 5) DSM-5 diagnosis of MAUD. The exclusion criteria were: 1) other lifetime/current illicit SUDs such as heroin and ketamine (except for alcohol and tobacco), 2) unable to understand research content, 3) diagnosis of so-called organic brain diseases or severe mental disorders (e.g., schizophrenia, major depression, bipolar disorder).

We recruited a gender-, age-, and education-matched healthy control group that was drawn nationally from a mobile phone survey conducted between January 2022 and February 2022. The control group comprised of healthy participants aged between 18 and 60 years with no history of mental illness or SUD (except possibly for tobacco). Individuals with severe physical or mental illness that could impair cognitive performance or inability to read Chinese and give informed consent were excluded from both groups. We screened a total of 376 MAUD patients and 905 healthy control subjects and finally recruited 360 eligible MAUD patients and 604 eligible healthy control subjects who completed the study, providing valid questionnaires based on completion time and quality of the survey answers.

The study was approved by the Ethics Committee of The Second Xiangya Hospital, Central South University. A semi-structured interview and self-reported questionnaire survey were administrated to each participant by two psychiatrists who validated the raters' consistency. All participants were provided information about the purpose and procedure of the study and were informed that they were free to quit at any stage of the study and any information we obtained was confidential, for research only. Informed consent to participate was obtained from each participant before they completed the study.

### Materials and Measures

#### Demographics and MA-Use Characteristics

Semi-structured interviews and questionnaires were used to collect demographics for both groups and MA-use characteristics for the MAUD group only. Demographic data included gender, age, education, marital status, and employment (full-time job, part-time job, or unemployment). MA-use characteristics included the age of first MA use, age of MAUD onset, duration of MA use (years from the age at first MA use to age at the time of enrollment), the longest period of MA abstinence (specifically referring to the longest voluntary MA detoxification/abstinence period rather than during compulsory treatment), MA-induced paranoia (a positive answer of the item: “Have you ever had a paranoid experience when you were using MA?”), the severity of MAUD (having six or more DSM-5 criteria for stimulant use disorder was considered severe MAUD, while two to five criteria was considered mild to moderate), co-occurring alcohol use disorder (AUD) [a score of eight or more on the Alcohol Use Disorders Identification Test (AUDIT) measured for the past year before entering the Compulsory Drug Rehabilitation Center (28, 29)] and co-occurring tobacco use disorder (TUD) [a score of six or more on the Fagerström Test for Nicotine Dependence (FTND) measured for the past year before entering the Compulsory Drug Rehabilitation Center (30, 31)].

#### Childhood Trauma

Childhood trauma was evaluated in both groups using the validated Chinese version of the Childhood Trauma Questionnaire (32) that was based on the short form of the Childhood Trauma Questionnaire (CTQ-SF) to measure traumatic experiences before the age of 16 (33, 34). It is a self-administered instrument that includes 28 items rated on a 5-point Likert-type scale ranging from 1 “never” to 5 “always.” The CTQ-SF assesses five aspects of traumatic experiences, including emotional abuse, physical abuse, sexual abuse, emotional neglect, and physical neglect. For the purpose of the current study, the total score was calculated, with higher scores reflecting more severe childhood trauma. In the current MAUD sample, the Cronbach's α of the questionnaire was 0.87.

#### Aggression

The severity of aggression was measured in both groups using the Chinese version of the Buss-Perry Aggressive Questionnaire (AQ-CV) (35) adapted from Aggression Questionnaire (AQ) developed by Buss and Perry (36) and also validated in individuals with MAUD (19, 37) to measure current level of aggression. The AQ-CV is a self-reported questionnaire of 30 items that assesses five domains of aggression: physical aggression (seven items), verbal aggression (five items), anger (six items), hostility (seven items), and self-aggression (five items), with each rated on a 5-point Likert scale of 1 “extremely uncharacteristic of me” to 5 “extremely characteristic of me.” The score of each sub-scale was the sum of the scores of the items contained, and then mathematically transformed to a centesimal score, with higher scores reflecting higher aggression. The total score of AQ-CV is the centesimal score of sum of all item scores and reflects participants' levels of overall aggression. In the MAUD sample in this study, the Cronbach's α for the AQ-CV was 0.92, and the Cronbach's α values for the five sub-scales ranged from 0.58 for verbal aggression to 0.80 for physical aggression and anger.

### Statistical Analysis

First, descriptive statistics were used to describe demographics, childhood trauma, and aggression for both groups, as well as MA-use characteristics for the MAUD group only. Each continuous variable was described by mean (M) and standard deviation (SD) for normal distributions. The categorical variables were described by numbers and proportions. Second, independent sample *t*-tests for continuous variables and Chi-square tests for categorical variables were used to compare the demographics and the severity of childhood trauma and aggression between the MAUD group and the control group. Third, simple linear regression analyses were used to initially identify relationships between aggression and demographics, MA-use characteristics, and childhood trauma in the MAUD group. Next, multivariate linear regression analyses were applied to identify independent relationships with aggression in the MAUD group. In light of the differences in the overall and five dimensions of aggression, we conducted six regression models with overall aggression, physical aggression, verbal aggression, self-aggression, anger, and hostility as dependent variables, and all significant variables in the previous univariate analyses as independent variables. All analyses in this study were executed in IBM SPSS Statistics 23.0 software, and the two-tailed significance level was set as *p* < 0.05. Of note, given the clinical relevance of MAUD onset, we designated age of MAUD onset instead of age and age of first MA use as the independent variable in the multivariate linear regression models to avoid multicollinearity.

## Results

### Sample Characteristics and Group Comparisons

Table 1 presents sample characteristics and group comparisons between the MAUD group and the control group.

**Table 1:** Sample characteristics and group comparisons.

**Variables**	**MAUD group**	**Control group**	**c**^**2**^/**t**	***p***
	***n*** **= 360**	***n*** **= 604**		
**Demographics**				
Male	360 (100)	604 (100)		
Age	33.65 (6.38)	33.27 (8.95)	−0.77	0.439
Junior high school or above	283 (78.6)	493 (81.6)	1.30	0.254
Married	159 (44.2)	446 (73.8)	84.98	<0.001
Full-time job	227 (63.1)	518 (85.8)	66.24	<0.001
**MA-use characteristics**				
Age of first MA use	26.49 (7.06)	–	–	–
Age of MAUD onset	30.00 (6.56)	–	–	–
Duration of MA use (years)	7.16 (3.57)	–	–	–
Longest period of MA abstinence (months)	12.52 (14.25)	–	–	–
MA-induced paranoia	145 (40.3)	–	–	–
Severe of MAUD	278 (77.2)	–	–	–
Co-occurring AUD	166 (46.1)	–	–	–
Co-occurring TUD	126 (35.0)	–	–	–
**CTQ-SF total score**	38.70 (11.83)	35.50 (9.57)	−4.34	<0.001
Emotional Abuse, EA	7.12 (2.59)	6.76 (2.45)	−2.14	0.032
Physical Abuse, PA	6.63 (2.93)	5.81 (1.89)	−4.75	<0.001
Sexual Abuse, SA	6.16 (2.30)	5.43 (1.31)	−5.51	<0.001
Emotional Neglect, EN	10.38 (4.97)	9.56 (4.53)	−2.54	0.011
Physical Neglect, PN	8.41 (3.41)	7.93 (2.87)	−2.21	0.027
**AQ-CV total score**	34.41 (14.98)	24.00 (14.66)	−10.58	<0.001
Physical Aggression, PAG	40.94 (20.20)	22.41 (16.32)	−14.77	<0.001
Verbal Aggression, VAG	38.46 (15.79)	28.28 (16.94)	−9.427	<0.001
Self-aggression, SAG	29.09 (17.99)	17.04 (16.70)	−10.53	<0.001
Anger, A	36.00 (20.19)	28.06 (20.39)	−5.87	<0.001
Hostility, H	27.43 (16.78)	24.01 (17.53)	−2.98	0.003

*MA, methamphetamine; MAUD, methamphetamine use disorder; AUD, alcohol use disorder; TUD, tobacco use disorder; CTQ-SF, the short form of the Childhood Trauma Questionnaire; AQ-CV, the Chinese version of the Buss-Perry Aggressive Questionnaire*.

The MAUD group was all male (100%), with a mean age of 33.65 ± 6.38 years (range: 19–53), predominantly with junior high school or above education (78.6%), not married (55.8%), and with full-time jobs (63.1%). Regarding MA-use characteristics, the MAUD group had a mean of 26.49 years for first MA use age, 30.00 years for MAUD onset age, 7.16 years for MA use duration, and 12.52 months for the longest period of MA abstinence. Most had severe MAUD (77.2%) and large minorities had MA-induced paranoia (40.3%), co-occurring AUD (46.1%), and co-occurring TUD (35.0%). The MAUD group had a total mean score of 38.70 for childhood trauma, with its five sub-scales ranging from 6.16 for sexual abuse to 10.38 for emotional neglect. The MAUD group had a mean score of 34.41 for aggression, with its five sub-scales ranging from 27.43 for hostility to 40.94 for physical aggression.

The matched control group had comparable ages and education levels with the MAUD group, but significant differences existed in other demographic characteristics, childhood trauma, and aggression. Compared to the control group, the MAUD group was less likely to be married (44.2% vs. 73.8%, *p* < 0.001), and have full-time jobs (63.1% vs. 85.8%). The MAUD group also scored higher on childhood trauma (38.70 ± 11.83 vs. 35.50 ± 9.57, *p* < 0.001) and aggression (34.41 ± 14.98 vs. 24.00 ± 14.66, *p* < 0.001), including on each sub-scale.

### Univariate Analyses

Table 2 shows the univariate analyses of the associations of demographics, MA-use characteristics, and childhood trauma with aggression in the MAUD group. The total aggression score was negatively associated with age (β = −0.14, *p* < 0.01), junior high school or above education (β = −0.13, *p* < 0.05), being married (β = −0.13, *p* < 0.05), age of first MA use (β = −0.13, *p* < 0.05), and age of MAUD onset (β = −0.18, *p* < 0.01). The total aggression score was positively associated with having MA-induced paranoia (β = 0.18, *p* < 0.01), severe MAUD (β = 0.21, *p* < 0.001), co-occurring AUD (β = 0.14, *p* < 0.05), co-occurring TUD (β = 0.23, *p* < 0.001), and the childhood trauma total score (β = 0.20, *p* < 0.001), as well as each of its five sub-scales (β = 0.14–0.17, *p* < 0.01). Slight differences existed in the associations of the five sub-scales of aggression with demographics, MA-use characteristics, and childhood trauma, with details shown in Table 2.

**Table 2:** Univariate analyses of aggression.

**Variables**	**Overall aggression**	**PAG**	**VAG**	**SAG**	**A**	**H**
**Demographics** ^**a**^						
Age	−0.14^**^	−0.19^***^	−0.05	−0.11^*^	−0.10	−0.09
Junior high school or above	−0.13^*^	−0.12^*^	−0.07	−0.11^*^	−0.11^*^	−0.11^*^
Married	−0.13^*^	−0.10	−0.04	−0.16^**^	−0.06	−0.15^**^
Full-time job	−0.02	−0.03	−0.01	−0.03	−0.05	0.02
**MA-use characteristics** ^**a**^						
Age of first MA use	−0.13^*^	−0.21^***^	−0.06	−0.09	−0.10	−0.04
Age of MAUD onset	−0.18^**^	−0.23^***^	−0.09	−0.14^**^	−0.14^**^	−0.10
Duration of MA use (years)	0.01	0.08	0.02	−0.02	0.01	−0.07
Longest period of MA abstinence (months)	0.02	0.01	−0.05	0.06	−0.01	0.04
MA-induced paranoia	0.18^**^	0.10	0.05	0.22^***^	0.15^**^	0.21^***^
Severe MAUD	0.21^***^	0.21^***^	0.12^*^	0.13^*^	0.20^***^	0.17^**^
Co-occurring AUD	0.14^*^	0.17^**^	−0.02	0.19^***^	0.05	0.14^*^
Co-occurring TUD	0.23^***^	0.24^***^	0.15^**^	0.21^***^	0.21^***^	0.12^*^
**CTQ-SF total score** ^**a**^	0.20^***^	0.15^**^	0.06	0.20^***^	0.18^**^	0.22^***^
Emotional Abuse, EA	0.17^**^	0.12^*^	0.06	0.19^***^	0.12^*^	0.19^***^
Physical Abuse, PA	0.15^**^	0.16^**^	0.04	0.13^*^	0.12^*^	0.14^*^
Sexual Abuse, SA	0.16^**^	0.12^*^	0.06	0.18^**^	0.11^*^	0.17^**^
Emotional Neglect, EN	0.14^**^	0.10	0.05	0.11^*^	0.16^**^	0.15^**^
Physical Neglect, PN	0.14^**^	0.08	0.03	0.15^**^	0.12^*^	0.16^**^

* *p < 0.05;*

** *p < 0.01;*

*** *p < 0.001.*

a *Standardized coefficient β*.

*MA, methamphetamine; MAUD, methamphetamine use disorder; AUD, alcohol use disorder; TUD, tobacco use disorder; CTQ-SF, the short form of the Childhood Trauma Questionnaire; PAG, physical aggression; VAG, verbal aggression; SAG, self-aggression; A, anger; H, hostility*.

### Multivariate Analyses

Table 3 shows the results of six multivariate linear regressions to determine independent factors linked to aggression and its five domains. Age of MAUD onset was negatively correlated with overall aggression, while having severe MAUD, co-occurring AUD, co-occurring TUD and childhood trauma were positively associated with overall aggression. Slightly different correlates were found for its five domains. Physical aggression was inversely associated with junior high school or above education and age of MAUD onset, and positively associated with severe MAUD, co-occurring AUD/TUD and childhood trauma. Verbal aggression was positively associated with severe MAUD and co-occurring TUD. Self-aggression was inversely associated with being married and positively associated with MA-induced paranoia, co-occurring AUD/TUD, and childhood trauma. Anger was positively associated with severe MAUD, co-occurring TUD and childhood trauma. Hostility was inversely associated with being married and positively associated with MA-induced paranoia, severe MAUD, co-occurring AUD and childhood trauma.

**Table 3:** Multivariate linear regression analyses of aggression.

**Aggression domains**	**Correlates**	**β**	***T***	***p***
Overall aggression				
	Age of MAUD onset	−0.11	−2.04	0.043
	Severe MAUD	0.15	2.80	0.005
	Co-occurring AUD	0.11	2.16	0.031
	Co-occurring TUD	0.18	3.55	<0.001
	Childhood trauma	0.15	2.91	0.004
Physical aggression				
	Junior high school or above	−0.12	−2.34	0.020
	Age of MAUD onset	−0.17	−3.39	0.001
	Severe MAUD	0.15	2.89	0.004
	Co-occurring AUD	0.13	2.59	0.010
	Co-occurring TUD	0.20	4.00	<0.001
	Childhood trauma	0.10	2.02	0.044
Verbal aggression				
	Severe MAUD	0.11	1.99	0.048
	Co-occurring TUD	0.13	2.50	0.013
Self-aggression				
	Married	−0.11	−2.29	0.023
	MA-induced paranoia	0.16	3.23	0.001
	Co-occurring AUD	0.16	3.28	0.001
	Co-occurring TUD	0.15	3.07	0.002
	Childhood trauma	0.14	2.71	0.007
Anger				
	Severe MAUD	0.14	2.64	0.009
	Co-occurring TUD	0.17	3.40	0.001
	Childhood trauma	0.14	2.73	0.007
Hostility				
	Married	−0.12	−2.39	0.017
	MA-induced paranoia	0.14	2.78	0.006
	Severe MAUD	0.13	2.53	0.012
	Co-occurring AUD	0.13	2.63	0.009
	Childhood trauma	0.17	3.31	0.001

*MA, methamphetamine; MAUD, methamphetamine use disorder; AUD, alcohol use disorder; TUD, tobacco use disorder*.

## Discussion

### Childhood Trauma and Aggression in MAUD

Our findings showed higher levels of childhood trauma and aggression in the MAUD group than the matched healthy control group. The higher level of childhood trauma in the MAUD group was consistent with previous studies showing high childhood trauma among people who use MA (38) and exposure to childhood trauma being related to subsequent initiation of MA use (39, 40). Childhood trauma has been established as an important factor linked to substance abuse including MA abuse (10, 41). Exposure to childhood trauma may increase vulnerability to emotional distress and mental disorders, and people with childhood trauma may resort to substance abuse as a coping mechanism to deal with psychological impacts of childhood trauma (42, 43). The higher level of aggression in the MAUD group was consistent with previous studies showing positive associations between substance abuse, aggression, and violence (44–46). The strong link between MAUD and aggression may be explained by multiple factors, including altered brain function, decreased ability to inhibit impulsive behaviors, or impaired social cognition, among others (8).

### Associations Between Childhood Trauma and Aggression

Results from the study generally support positive associations between childhood trauma and aggression, reflected in all aggression sub-scales except for verbal aggression. This finding was consistent with previous studies showing childhood trauma as a factor linked to adult aggression in other populations such as prisoners (20), college students (21), and the general population (22). This result also aligns with proposed mechanisms linking childhood trauma to aggression through substance abuse (26). Experiencing childhood trauma may lead to significant changes in brain structure and neuroendocrine function, such as decreased gray matter volumes of the caudate nucleus, hippocampus, and amygdala, and lower cortisol levels during arousal (47, 48), promoting emotional disorders and aggression. Our findings suggest that childhood trauma may constitute an independent, significant, and important risk factor for aggression among patients with MAUD, even after controlling for MA-use characteristics and demographics. Thus, special attention may be needed to address coping with childhood trauma when designing intervention programs targeted at limiting aggression among patients with MAUD.

### Relationships Between Demographics and MA-Use Characteristics and Aggression

Our study found significant associations between a range of MA-use characteristics, certain demographics and aggression among patients with MAUD. To be specific, the age of MAUD onset, having severe MAUD, and co-occurring AUD/TUD were associated with overall aggression, while other factors including having MA-induced paranoia, junior high school or above education and being married were associated with specific dimensions.

The negative association between the age of MAUD onset and aggression was consistent with prior studies showing that younger age of MA use and MAUD onset were associated with aggression, especially physical aggression (11, 12). Studies have shown that the early age of MA use and related MAUD onset increased the likelihood of engaging in MA-related violence, after adjusting for socio-demographics and other potential confounders (11). Additionally, age at first perpetration of violence against another person was positively associated with age at first use of MA, and with age at which the person regularly used MA (12). Therefore, when treating and managing patients with younger onset ages of MAUD, special attention may need to be given to assessing and preventing physical aggression.

Although previous studies have shown that chronic MA use (13) and more frequent MA use (14) were associated with aggression, our study failed to identify such associations. Instead, multivariate regression analyses showed a higher level of aggression among patients with severe MAUD than those with mild to moderate MAUD. These findings may reflect greater MA-use severity associated with chronic and more frequent use rather than duration and frequency of MA use *per se*. One study found a MA dose-dependent increase in aggressive behavior, with the risk probability of aggression increasing from 9 to 52% in relation to various degrees of MA use ranging from abstinence to heavy use (49). Our findings add further evidence to the potential dose-response relationship between MA-use severity and aggression in MAUD.

The finding that co-occurring AUD/TUD was associated with aggression among patients with MAUD was consistent with previous studies showing links between alcohol and tobacco use and aggression (19, 50, 51). For instance, a study showed that simultaneous alcohol and MA use was associated with elevated odds of MA-related aggression and hostility, with an adjusted odds ratio of 2.74 (19). Moreover, MA, alcohol, and tobacco all may affect cognitive functioning and increase the likelihood of perceiving an environmental stimulus as threatening, potentially leading to impulsive and aggressive responses to the perceived threats (19). Our findings suggest that co-occurring AUD/TUD were common and associated with aggression among people with MAUD, and we believe that this warrants further attention in clinical settings.

Our study also found other MA-use characteristics and demographics associated with specific dimensions of aggression. Compared with patients without MA-induced paranoia, patients who have experienced MA-induced paranoia showed a higher degree of self-aggression and hostility. This finding was in keeping with previous studies showing positive associations between positive psychotic symptoms (e.g., hallucinations and paranoia) and hostility (52) and self-aggressive behaviors including suicide (53). These findings suggest that paranoia is an important factor linking MAUD with aggression that warrants particular attention. Regarding demographics, our study found junior high school or above education and being married to be possible protective factors mitigating against physical aggression, self-aggression and hostility, in line with prior studies (54–56). The link between education and aggression may be bidirectional; patients with higher educational attainment may have better health literacy and resources to control aggressive tendencies, while those with aggressive behaviors may be at increased risk of poor school performance, thus leading to lower academic achievement (57). The findings speculatively suggest that strengthening education among MAUD patients may help with the prevention and reduction of aggression. Regarding marital status and aggression, an explanation may be that individuals showing higher levels of hostility and self-aggression were more likely to experience dissolution or termination of their relationships as a result of their aggression. The potentially bidirectional relationship between marital status and aggression should be further tested in future longitudinal study designs.

### Limitation and Strengths

Several limitations warrant consideration. First, we used a convenience sample from a compulsory drug rehabilitation center in Changsha City of Hunan Province in China, which may not represent other MAUD populations from other organizations or from the community and in other areas of China. Future multi-center studies that include MAUD populations from various organizations and communities in various parts of China are needed to get a more representative sample. Second, we only included male MAUD patients in our study since aggression is much more common among male MAUD patients, and all MAUD patients in the Compulsory Drug Rehabilitation Center in our study were male. Future studies should include both male and female MAUD patients to examine gender differences of aggression among MAUD patients. Third, the cross-sectional nature of the study precluded making causal inferences. Future longitudinal studies are needed to examine temporal relationships. Fourth, the extent to which specific aspects of aggression link to specific sample characteristics warrants study in larger samples as strengths of between-domain relationships were not tested statistically. Fifth, data collection of certain questionnaires such as the Childhood Trauma Questionnaire is based on recall of traumatic childhood experiences that require full cognitive function. MAUD patients may have cognitive impairments that affect their accurate memories of past experiences and thus affect the accuracy of data collection quality. Future research may consider assessing and controlling for the cognitive function of the MAUD patients to reduce such potential recall bias. Finally, we failed to find any interaction (moderation and/or mediation) between childhood trauma and all eight selected MA-use characteristics on aggression in our study (results not shown here). On the one hand, this may indicate that both childhood trauma and MA-use characteristics work independently on aggression. On the other hand, this may imply that childhood trauma affect aggression through other MA-use characteristics not included in the current study. Future studies are needed to further explore potential underlying mechanism of childhood trauma on aggression.

Despite these limitations, our study is the first to our knowledge to compare childhood trauma and aggression between MAUD and control groups and to explore various dimensions of aggression among male patients with MAUD in China. The inclusion of a control group helps to better understand associations between MAUD, childhood trauma, and aggression. Exploration of various dimensions of aggression facilitates a better understanding of specific relationships. Furthermore, this study examined the associations between a wide range of potential risk factors and aggression among patients with MAUD. These factors were selected based on previous studies with a solid theoretical background and included mainly MA-use-related characteristics (age, duration and frequency of MA use, psychotic symptoms, and concurrent alcohol and tobacco use) and childhood trauma (emotional abuse, physical abuse, sexual abuse, emotional neglect, and physical neglect). Our study observed positive associations of both MA-use characteristics and childhood trauma with aggression among patients with MAUD. As China is the most populous country worldwide and has prevalent MAUD, the findings have implications for a large group of people and expand the literature beyond WEIRD (White, educated, industrialized, rich, and democratic) countries (58). The extent to which the findings extend to other jurisdictions warrants direct examination.

## Conclusions

This study showed higher levels of childhood trauma and aggression in MAUD patients than in healthy control subjects from the general population. Both MA-use-related characteristics (mainly age of MAUD onset, severe MAUD, and co-occurring AUD/TUD), and childhood trauma were associated with aggression among patients with MAUD. The results provide novel and useful information on potential risk factors for aggression and its various dimensions and inform future longitudinal research to establish causal relationships between these factors and aggression to guide further prevention and treatment programs on aggression.

## Data Availability Statement

The data supporting the conclusions of this article will be made available by the corresponding authors, with any reasonable reasons. Requests to access the data should be directed to WH, weihao57@csu.edu.cn.

## Ethics Statement

This study was approved by the Ethics Committee of the Second Xiangya Hospital, Central South University. All participants understood the purpose and procedure of the study and provided their informed consent.

## Author Contributions

WH and TL designed the study. XZ supervised the whole process. HS and QD provided support for data collection of MAUD group. ML and YW performed the semi-structure interviews and data collection for MAUD group with the participation of CY and TF. LP collected healthy control group data. WL and XF assisted in the data analysis and interpretation. ML managed the literature search and drafted the manuscript. MP directed the manuscript and revised it. All authors contributed to the article and approved the submitted version.

## Funding

This study was supported by grants from the Key Program of the National Natural Science Foundation of China (81130020, WH), the National Natural Science Foundation of China (30971050, WH), the National 973 Program (2015CB553500, WH), and the National Key R&D Program of China (2017YFC1310400, TL).

## Conflict of Interest

The authors declare that the research was conducted in the absence of any commercial or financial relationships that could be construed as a potential conflict of interest.

## Publisher's Note

All claims expressed in this article are solely those of the authors and do not necessarily represent those of their affiliated organizations, or those of the publisher, the editors and the reviewers. Any product that may be evaluated in this article, or claim that may be made by its manufacturer, is not guaranteed or endorsed by the publisher.
